# The Role of the Multidisciplinary HIV Care Center in Mitigating Social Isolation Among Patients with HIV During the Early COVID-19 Pandemic

**DOI:** 10.1007/s10461-024-04395-y

**Published:** 2024-06-10

**Authors:** Ofole Mgbako, Claire Loughran, Michael P. Vaughn, Jason Felder, Ashley Augustin, Peter Gordon, Robert H. Remien, Susan Olender

**Affiliations:** 1 Division of Infectious Diseases and Immunology, Department of Internal Medicine, NYU Grossman School of Medicine, 462 1st Ave. NY, New York, NY 10016 USA; 2 NYU Langone Institute for Excellence in Health Equity, New York, NY USA; 3 NewYork Presbyterian, Columbia University Irving Medical Center, New York, NY USA; 4 HIV Center for Clinical and Behavioral Studies, NY State Psychiatric Institute and Columbia University, New York, NY USA; 5 Division of Infectious Disease, Department of Internal Medicine, Columbia University Irving Medical Center, New York, NY USA

**Keywords:** HIV, COVID-19, Social isolation, Care engagement

## Abstract

As the COVID-19 pandemic began in 2020, significant public health mitigation efforts were vital to combat an unprecedented health crisis. These efforts, which involved social distancing and self-quarantine, likely worsened a public health crisis of social isolation and loneliness in the U.S., particularly among people with HIV (PWH). Multidisciplinary HIV care centers, which served as the main source of clinical care for PWH and in some cases the only point of social contact, faced evolving dynamics of in-person visits during the COVID-19 pandemic, as well as a shift to telehealth services. Using in-depth interviews, we explored the role that multidisciplinary HIV care centers and providers played in the experience of social isolation among PWH in New York City. We recruited participants (*n* = 30) from a multidisciplinary HIV care center in NYC between October 2020 and June 2021. We conducted semi-structured interviews to understand the specific domains of social isolation that were mitigated. In this cohort, the major theme that drove both in-person and telehealth care continuity was the strength of the patient-provider relationship. We found that participants saw members of the HIV care center as part of their social network, and providers served both as a source of emotional support and provided important social resources and benefits. Thus, in times of heightened social isolation, HIV care centers can play a critical role in providing social support in addition to clinical care.

## Introduction

As the COVID-19 pandemic began in 2020, significant public health mitigation efforts, including social distancing and quarantine were required, particularly for the most socially and medically vulnerable in society. Levels of social isolation, already a public health issue before COVID-19, subsequently rose dramatically in the United States [1, 2]. The effects of social isolation on mental and physical health, as well as mortality, have been well documented in the literature and recognized by the U.S. Surgeon General and other authorities as a public health crisis [3–5]. People with HIV (PWH) often have heightened vulnerability to social isolation compared to the general population due to fear of diagnosis disclosure to family and friends or aversion to forming new sexual or romantic relationships due to stigma and the real prospects of societal discrimination [6, 7]. These complex social and behavioral dynamics post-HIV diagnosis are important to understand in the context of the COVID-19 pandemic.

During the COVID-19 pandemic, the evolving nature of in-person visits for HIV clinical care – such as the altered workflow around waiting rooms and the need for masking during clinical encounters – as well as the move towards telehealth, led to important changes in HIV clinical care delivery. Furthermore, during the period of enforced social isolation in the early COVID-19 pandemic, multidisciplinary HIV care centers sometimes served as the only point of in-person social contact for PWH. Adequate social support and feelings of connectedness for PWH has been associated with HIV care retention [8] and the size of one’s social network has been associated with better quality of life for older PWH [9]. Exploring patient experiences of the early COVID-19 pandemic on both social isolation and HIV care engagement is essential.

While there is a vast literature on the role of multidisciplinary HIV care centers in promoting retention in HIV care and the elements that influence successful HIV care engagement, such as high-quality communication and trusting relationships with providers [10, 11], there is a paucity of studies in the literature on the role of multidisciplinary HIV care centers in mitigating feelings of social isolation among PWH. Social isolation has multiple dimensions, including the number of personal connections, the overall structure of one’s social network, and the quality of those relationships. There is no agreed upon conceptual framework for social isolation in the literature, however Wang et al. describes five different domains to consider: (1) social network – quantity, (2) social network – structure, (3) social network – quality, (4) appraisal of relationships – emotional, and (5) appraisal of relationships – resources [12].

As part of a larger observational pilot study, we employed a qualitative approach to explore the barriers and facilitators to care continuity during the early COVID-19 pandemic. For this study, we conducted a secondary analysis to explore the role that multidisciplinary HIV care centers and providers played in mitigating social isolation among PWH in New York City (NYC) during the same period. We hypothesized that the clinical environment, as well as providers (i.e., multidisciplinary teams, including social workers, care coordinators, community health workers, and nurses) were critical in mitigating social isolation among this vulnerable population. Utilizing the framework in Wang et al., we sought to explore which specific domains of isolation were mitigated by in-person visits and telehealth visits.

## Methods

### The Setting

Our comprehensive multidisciplinary HIV care center is part of a large academic medical center serving a racially and ethnically diverse community in NYC. During the early months of the COVID-19 pandemic, the center remained open for urgent or walk-in care given the number of patients facing adverse social determinants of health, such as homelessness or unemployment, and needing in-person support to safely stay in care and on treatment. The HIV care center also opened and scaled up in-person clinical services earlier than other similar sites in NYC. Telehealth services were preferentially used for patients who had access to technology and reliable internet.

### The Multidisciplinary HIV Care Model

The multidisciplinary care center practices in a team-based format in which teams care for a shared panel of patients. The “team” includes medical providers who provide HIV and general primary care, a behavioral health clinician (licensed social worker), case managers, a community health worker and a nurse care manager. Teams meet weekly for practice panel management and focus on key quality indicators such as viremia, untreated hepatitis C or missed visits. Care is organized as an Open Access model which encourages low threshold care for same day encounters. All medical providers have reserved Open Access slots on any given day/week. Patients are outreached and re-engaged in care if they miss visits and are invited either to come in immediately or within 7 days as part of Open Access care and panel management. Case managers and community health workers routinely outreach patients who are missing visits by phone or through home visits.

### Study Design

As a part of the larger observational study, a thematic analysis of semi-structured interviews was conducted using an inductive approach. Within the larger study, major themes were identified to address participant perceptions of rapid or immediate antiretroviral therapy and its impact on HIV care engagement, highlighting barriers and facilitators to HIV clinical care during COVID-19. For this secondary analysis, we conducted an additional thematic analysis using a deductive approach guided by Wang’s conceptual framework to understand how our multidisciplinary HIV care centers mitigated social isolation.

### Participants and Eligibility Criteria

We recruited participants (*n* = 30) from a multidisciplinary HIV care center in NYC between October 2020 and June 2021. Inclusion criteria were: (1) aged 18 years or older; (2) diagnosed with HIV on or after January 1, 2018; (3) enrolled in care for at least 6 months; (4) residing within one of the NYC boroughs; and (5) English or Spanish-speaking.

### Demographic Survey

Participant demographics collected included age, country of birth, primary language spoken, race/ethnicity, gender identity, sexual orientation, education level, employment status, housing situation, annual income, health insurance and relationship status.

### Procedures

Participants were recruited in person and over the phone by providers, social workers, and care coordinators, along with flyers posted in clinic waiting areas. With verbal agreement, participants were contacted by phone and electronically consented via REDCap using an IRB-approved consent form.

Due to the on-going COVID-19 pandemic, in-depth interviews were conducted either in person or via Zoom, based on the participant’s preference. Of the 30 people enrolled, 20 participated virtually, and 10 participated in-person. We conducted 4 interviews in Spanish and 26 in English. To ensure fidelity between languages, the interview guide was translated and back-translated by a native Spanish-speaker. Interviews lasted 60–90 min and were audio-recorded. Audio recordings were transcribed verbatim for analysis, with Spanish-language transcriptions professionally translated to English. Participants received $50 for the completion of the study activities via electronic gift cards.

### Qualitative Interview Guide

We conducted one-on-one in-depth interviews using a semi-structured interview guide iteratively developed by the research team. After piloting the interview guide with the first two participants, appropriate adjustments were made to ensure the meaning and intention of questions were understood. The data gathered from these participants was included in the final analysis. The interview guide from the larger observational study included five domains that answered the parent study research question; however, this analysis focuses specifically on data from two of the five domains: (1) social support and (2) COVID-19’s impact on patients’ HIV care and clinic engagement. Probes were utilized to elicit more information as needed.

### Social Isolation Framework

Wang et al. sought to provide conceptual clarity to the different domains of social isolation by presenting an organizing framework separated into the 5 aforementioned domains [12]. Each domain is further defined below and was applied to our qualitative results:

1. Social network – quantity: *refers to the number of people in someone’s social network*.
2. Social network – structure: *refers to network density and characteristics (e.g. how many people in the network also know each other, how many are family or health professionals)*.
3. Social network – quality: *refers to perceived quality of relationships*.
4. Appraisal of relationships – emotional: *refers to overall appraisal of perceived adequacy or impact of relationships*.
5. Appraisal of relationships – resources: *refers to overall appraisal of perceived access to resources through one’s social relationships (i.e. access to information)*.

### Data Analysis

Development of an initial code book by authors OM and CL, trained in qualitative data analysis, using a priori codes guided by the conceptual framework and emergent codes. OM and CL independently coded transcripts and resolved inconsistencies with the research team and the development of the final codebook. Inter-coder agreement calculations found a pooled Kappa of 0.91. Dedoose was used to analyze the data, looking for code co-occurrence pertinent to our research question. We then grouped coded segments into categories, allowing themes to emerge for our inductive approach and grouping coded segments into Wang et al.’s pre-existing framework for our deductive approach. Our analytic approach emphasized the core tenet of *maximum variation*, or identifying the widest array of experiences possible, sometimes referred to as *saturation* [13]. As such, our approach required a deep dive into individual experiences, rather than a focus on the commonality of these experiences, in hopes that these rich findings can provide clarity for present-day clinicians. We took each participant’s story as a complex narrative, across which themes emerged.

### Ethics

Clinicians and non-clinicians conducted the data collection and analysis for this paper. We used subjectivity memos, which are real-time self-reflective notes written by the interviewer, to address researcher bias based on the interviewer’s position to the subject matter. The study was approved by the Columbia University Irving Medical Center Institutional Review Board. All participants gave informed written informed consent for study activities.

## Results

Participant demographics of the cohort are summarized in Table 1. Participant mean age (SD) was 35.4 (11.6). Most (80%) were cisgender men. Over half (53.3%) identified as gay, while 33.3% identified as straight and 6.7% identified as bisexual. About 33.3% were Black African/American and 60% were Hispanic. 36.6% were unemployed, 46.7% were on Medicaid, and 83.3% were never married, divorced, or separated. 83.3% had sustained viral suppression at 1 year.

**Table 1:** Demographics of research participants (N = 30)

		Total (N = 30)	
Characteristic		N (%)	
Age			
Mean (SD)		35.4 (11.6)	
Country of Birth			
United States		20 (66.7)	
US Territory		3 (10.0)	
Other		7 (23.3)	
Primary Language Spoken			
English		22 (73.3)	
Spanish		7 (23.3)	
French		0 (0.0)	
Haitian		0 (0.0)	
Other		1 (3.3)	
Race			
Black/African American		10 (33.3)	
Native American/ Alaskan Native		1 (3.3)	
White		3 (10.0)	
Other*		16 (53.3)	
Ethnicity			
Hispanic		18 (60.0)	
Non-Hispanic		12 (40.0)	
Gender Identity			
Cisgender Man		24 (80.0)	
Cisgender Woman		5 (16.7)	
Transgender Woman		1 (3.3)	
Sexual Orientation			
Heterosexual/ Straight		10 (33.3)	
Bisexual		2 (6.7)	
Gay		16 (53.3)	
Queer		1 (3.3)	
Unsure/Questioning		1 (3.3)	
Highest level of Education			
Less than High School		0 (0.0)	
Some High School		2 (6.7)	
High School Diploma/GED		6 (20.0)	
Some College		11 (36.7)	
Associate’s Degree		1 (3.3)	
Bachelor’s Degree		9 (30.0)	
Master’s Degree		1 (3.3)	
Employment Status			
Employed for wages		12 (40.0)	
Self-Employed		2 (6.7)	
Unemployed and looking		7 (23.3)	
Unemployed and not currently looking		4 (13.3)	
Student		2 (6.7)	
Retired		1 (3.3)	
Unable to work		2 (6.7)	
Housing Situation			
Stably Housed		28 (93.3)	
Unstably Housed		2 (6.7)	
Current Annual Income			
<$20,000		13 (43.3)	
$20,000–39,999		6 (20.0)	
$40,000–59,999		8 (26.7)	
$60,000–79,999		1 (3.3)	
>$80,000–99,999		2 (6.7)	
Health Insurance			
ADAP		4 (13.3)	
Medicaid		14 (46.7)	
Other		11 (36.7)	
None		0 (0.0)	
Don’t Know		1 (3.3)	
Relationship Status			
Never Married		19 (63.3)	
Divorced		3 (10.0)	
Separated		3 (10.0)	
Other		5 (16.7)	

*All participants who identified as other race identified as hispanic ethnicity

### Levels of Social Isolation

#### “If Push Comes to Shove, HIV Is the Least of My Worries”

Nineteen of the 30 participants expressed experiencing some form of social isolation within the context of the COVID-19 pandemic. Participants discussed how COVID-19 exacerbated feelings of isolation already present given challenges coping with their HIV diagnosis. One participant reported:

“I really have yet to share my diagnosis with my immediate family. The only people who know is the clinic where I go. I was homeless, living in the shelter. And I suffer from deep depression. I just wanted to end my life. And when I got diagnosed [with HIV] it was like a wakeup call…The [clinic] pulled me out of the hole that I was in. I have my nutritionist and she’s awesome. And a case worker and a social worker. I trust them a lot…Because of COVID it’s got even worse, you can’t even get to see a doctor. That scares me. I have other health issues, not just HIV. If push comes to shove, HIV is the least of my worries. In the beginning, when we were literally locked in and we couldn’t go out at all, I was terrified. I live alone and far from my kids. And my phone couldn’t Zoom because I have an old phone. I was paranoid. My children had to calm me down. Because of my depression, [I thought] I am going to get worse. And the COVID isn’t going to kill me, my depression is…. I didn’t talk to anyone, I didn’t go up to anybody, I didn’t shake hands. Nothing. But I did walk every day.” (Participant 1, 63 years, cisgender woman, Other race, Hispanic/Latino, *Appraisal of relationships - emotional*).

This participant expressed fear that the impact of social isolation brought on by both HIV and COVID-19 could worsen her mental health and even lead to her death. Without being able to disclose her HIV to family, the different members of the multidisciplinary care team (social worker, nutritionist, case worker) served as emotional support after her HIV diagnosis. Yet COVID-19 presented an unprecedented challenge. Several participants discussed their initial fear that the COVID-19 pandemic would interfere with their ability to tap into the social dimension of going to the care center, which for many was an important lifeline. Participant 23 also expressed this when she said:

“[During COVID-19], I had nobody. I had no one and I started to get a little depressed.” (Participant 23, 28 years, cisgender man, Black/African American, Hispanic).

The elements of the multidisciplinary care center that mitigated the potential of worsening social isolation for many participants are presented in the following thematic findings.

### Thematic Findings

#### “It’s Him That Keeps Me There”: The Power of the Patient-Provider Relationship in Both In-Person Visits and Telehealth During Early COVID-19

The early months of the COVID-19 pandemic impacted the ways in which most of our participants engaged in HIV care, primarily by creating workflows that minimized patients or staff congregating in areas of the care center and shifting services from in-person to telemedicine. For example, in spring of 2020 patients were no longer allowed to congregate in the waiting room and instead they were screened for COVID-19 symptoms at the care center entrance by a staff member in full personal protective equipment and immediately placed in a care center room. Participants regularly discussed it being potentially unsafe for them to attend in-person appointments due to risk of COVID-19 infection. A participant explained the perceived challenge of attempting to visit the HIV care center safely:

“Actually, at first, I had to come over [to the clinic] and there were a lot of restrictions taking place. Most of the time the clinic wasn’t closed down, but [COVID] did make things difficult for me, because I still had appointments. [There were] swabs going up the nose, every time you had an appointment you gotta throw your mask off and get your oxygen level tested…I did catch the symptoms [of COVID] eventually” (Participant 30, 72 years, cisgender man, Black/African American, Non-Hispanic/Latino).

Participants described the experience of going to the care center as difficult and fraught with fear of catching the virus at the start of the COVID-19 pandemic. Complicated logistics and new, evolving public health restrictions changed the experience of coming in for a regular appointment or bloodwork. However, most participants discussed good HIV care continuity despite pandemic-related uncertainty. For example, when asked about what had changed since the onset of the COVID-19 pandemic, and care prior to the pandemic, one participant reported:

“The only change was at the beginning when I couldn’t go into the clinic, and I had to do three or four visits over camera, over zoom. I usually go in and am able to sit down and chat with Dr. X or show her it hurts here or hurts there. It was not the same through a camera. It was a little more difficult explaining to her…I think I’ve gotten used to the zoom calls. So, I’ll do it. I usually do the zoom calls with her and she’ll submit any lab works that I need to get. And I’ll just walk in and do it really quick. But yeah, that’s the only difference with COVID, but I felt like I have continued receiving the same level of care from my doctor, from the clinic.” (Participant 14, 34 years, cisgender man, Other “Hispanic/Latino”, Hispanic/Latino, *Appraisal of relationships - resources*).

This participant gestures to the most common experience of virtually all participants in our cohort – the felt absence of in-person connection with their providers, even though the quality of care was maintained.

Another participant, however, described how in-person care was actually improved given restrictions around the number of people who could be in the care center at any given time. He described the dynamic in this way:

“I’ve had most of my appointments online, but when I do go into the office, I’ve gone in and it’s been very minimal people at the clinic. I have gone and gotten my blood work done. Sometimes I was the only person there, which is great. Overall, I still feel just as taken care of as I was before. Actually, I would have to say that I feel even a little bit more taken care of because it’s not like you’re waiting in the office.” (Participant 9, 29 years, cisgender male, White, Non-Hispanic/Latino).

Participants were generally highly engaged and had a very positive evaluation of the care center and their providers. However, another participant discussed their disrupted HIV care upon being discharged from the hospital after receiving a new HIV diagnosis:

“[After discharge] all departments I called said, “Oh, we not taking calls.” It was an unplanned pandemic, but there was no plan in place. What do you do? You know, with patients being treated or who need to be treated. I got a list of referrals on discharge but nobody answered when I called. They said I didn’t have a virtual platform, but they didn’t enroll me into the virtual platform so I was out of sync with the providers.” (Participant 16, 50 years, cisgender man, Black/African American, Non-Hispanic/Latino).

This same participant shared the challenges of finding HIV care after being discharged from the hospital during early COVID-19, and how it interrupted his ability to link to a new HIV care center. He goes on to explain the emotional connection he developed with his provider, and how it addressed some of these concerns:

“… I had so many problems [at the beginning of COVID]. If [my doctor] wasn’t my primary I would have given them the finger and walked away. It’s him that keeps me there. I say that because he’s thorough, he reaches out, he checks on me, ‘hey how’s that medication going?’ And that’s important. He doesn’t just prescribe it, he has a follow up system, to make sure I’m not getting nauseous or experiencing some allergic reaction or something. I’ve been in the hospital system for years and I worked with some awesome doctors but I haven’t met five of them like him” (Participant 16, 50 years, cisgender man, Black/African American, Non-Hispanic/Latino, *Appraisal of relationships - emotional*).

He states that even though he was overall very frustrated with his care, his relationship with his new HIV doctor encouraged him to continue antiretroviral treatment and stay in care. In this way, negative experiences due to COVID-19 related disruptions were mitigated by a strong, positive relationship showing that even a single provider may have the power to keep patients engaged.

Given the ongoing pandemic, telehealth served as a critical tool to ensure continuity of care. When discussing their HIV care, participants acknowledged the ways in which their healthcare teams provided *social support* and how telemedicine provided care continuity (e.g., access to medications, lab work, and medical advice) when in-person care was not safe amidst the COVID-19 pandemic. Another participant, when asked about COVID-19 related interruptions to their HIV care, shared that his experience with care continuity via telehealth was smooth:

“They were always there to provide attention. As I told you before, as the virus progressed and the city closed, they forbade in-person appointments. Phone calls, video calls, the app, any lab test results were on time. So, no. It didn’t affect me at all (Participant 7, 25 years, cisgender man, Other Race, “Latino,” Hispanic/Latino).

While our care center never officially closed to accommodate those who did not have telehealth capability and needed to be seen (many patients were not aware of this), this participant acknowledges what was consistent across the cohort – that telehealth served as an effective adjunct for continuity when in-person care was not advised. Some participants did express frustration with telehealth due to its inability to completely make up for in-person connection, but understood it as a temporizing measure. While participants experienced inconveniences during the early pandemic – whether from anxiety due to the perceived risk of COVID-19 acquisition during in-person visits, or the loss of nuance provided by the physical exam with telehealth care, or the lack of linkage support after a new diagnosis – these were overcome by heightened provider attention and strong patient-provider relationships.

### Emotional Connections and Resources: The Ways a Multidisciplinary HIV Care Center Mitigated COVID-19 Related Social Isolation

In the context of COVID-related social isolation, participants discussed positive emotions related to relationships with HIV care center providers during a very uncertain time. One participant shared the experience of seeing his provider again when discussing their first in-person appointment after COVID-19 began:

“COVID-19 has been a very, very big change for the world. I obviously could no longer go to the office when it was at its height. [I wanted] to talk to somebody. Just getting a regular checkup was only online. It did become a little difficult and just challenging overall. But we got through it. I saw [my doctor] last month. And it was good to know that she was okay. And, you know, catching up with her and stuff like that. It felt normal to just get bloodwork done and stuff like that. It was just really nice to see a familiar face again. And, just continuing with my medical care (Participant 21, 20 years, cisgender man, Other - “Latino,” Hispanic/Latino, *Appraisal of relationships – emotional*).

He poignantly remarked that it was “good to know that [his provider] was okay” when they had their first in-person appointment and remarked that it was “just really nice to see a familiar face again,” underscoring the importance of mutual patient-provider relationships built over time and the power of the familiarity of providers and the clinical environment, beyond just the access to care. The level of early COVID-19 mortality in NYC put many frontline providers at risk which was likely distressing to patients. This participant worried about his providers during a public health emergency given the intimate role they play in his life. This sentiment, related to *social network – quality* and *emotional appraisal of relationship* domains, was shared across the participant cohort and emphasizes that face-to-face contact with one’s provider and the sense of normalcy that comes with it can create and sustain high quality and emotionally-supportive connections.

Multiple participants expressed that care center visits were more about high quality social relationships than concerns about care continuity. Rather than simply being sources of expert information and medical resources, HIV providers were discussed as *people the patients could trust*. For example, another participant, who was able to maintain in-person visits during the early COVID-19 pandemic, shared the following when asked about HIV care engagement:

“I don’t know about everyone else’s [situation], I was in a position where I could just go to the clinic twice a year. So, my medicine refills were there already at the pharmacy. [My doctor] sends them like six months at a time for me. So, I didn’t really have to worry about running low on medicine or anything like that. When I did go during COVID-19, the only difference was we were all wearing masks. The doctor was advising me to get vaccinated and educating me all about that and everything. I told him I’m not sure if I want to get vaccinated right now. Because of the trust and the bond that we developed, he was just educating me on the reasons why it’s good for me to get vaccinated being HIV positive. I will get vaccinated when I feel like I’m ready to.” (Participant 17, 32 years, cisgender man, White/Other Race - “Latino,” Hispanic/Latino, *Appraisal of relationships – resources*).

This participant did not worry about medication refills or care continuity; rather, he focused on the ways in which his HIV care team supported and educated him, especially relating to decisions around vaccination. This fits into the domain of *appraisal of relationship – resources*. He explains, it was “because of the trust and the bond that [he and his providers] developed” that he felt comfortable discussing vaccine hesitancy. The HIV care center, in this instance, is both providing social and emotional support and leveraging trusting relationships to provide resources (i.e. COVID-related care) above and beyond the scope of HIV care continuity.

When participants discussed their relationships with providers, they often attributed the positive emotional valence to the providers acknowledging their whole personhood. For example, a participant mentioned the candor with which she could discuss COVID-19 with her providers and how it allowed her to discuss concerns about vaccines in the context of anti-Black racism in medicine. She first discussed how the COVID-19 vaccine made her feel uncomfortable because it reminded her of the medical experimentation on Black people in the US. However, she shared that he felt comfortable bringing this up to the nurses at the care center.

“We were taught very young in my household about the Tuskegee experiment and how Black men were injected with placebos… Well, I feel like when I go to the clinic, I see familiar faces and I am able to greet everyone and say hello to the nurses. And to speak candidly, even about this COVID situation. Like are they going to take the vaccine. Some of the nurses at my clinic said, I’m not taking the first batch. I’ll wait until the second trial. And I’m like how are you supposed to do that, isn’t it mandatory for you to take it? But I can understand their fear, because I have the same fears. So just to be able to speak candidly and openly with them, with everyone there. I like that.” (Participant 2, 43 years, cisgender woman, Black/African American, Non-Hispanic/Latina, *Appraisal of relationships – resources*).

This participant described the positive experience of feeling seen and having fears validated by care center staff. This experience underscores the capacity of in-person visits to tap into multiple domains of social support – *both resources from relationships and quality of interactions* – to facilitate challenging conversations about institutional racism within medicine. In the example she shared, she was given space to reflect on her own feelings of trust/mistrust of the medical system and vaccine decision-making, without judgement, guided by the nurse provider.

While telehealth was generally regarded as more of a supplement to in-person care, or a way to retain care continuity, some participants discussed the social benefit of telehealth relationships with providers. One participant, recounting a series of virtual interactions related to COVID and the COVID vaccine, shared the following:

“I’ll never forget messaging Dr. X. I was like, hey, I’m thinking about that COVID vaccine and she was like, we’re not giving it out at the clinic, but I’ll let you know. I ended up getting the vaccine in February, and then like a week later. Dr. X hit me up on the little connect. She was like, you were saying, you wanted that vaccine. We have it now. We can give it to you. And unfortunately, I had already gotten it. But the fact that she remembered that I had asked for it and just keeping up like that [was important] (Participant 18, 38 years, cisgender man, Black/African American, Non-Hispanic/Latino, *Social Network – quality*)”

This participant is clear that, even though he had already received the vaccine, his provider remembering and following up with him had a significant emotional impact, so much so that he says “I’ll never forget.” While this experience was more commonly discussed in terms of in-person care, telehealth also offered a platform for providers to build trusting and emotional relationships. As participants discussed, being able to safely remain in contact with their care providers (*network quality*) and maintain continuity of care were of utmost importance. The capacity to maintain and leverage social networks over a virtual platform underscores the potential of telehealth.

Another dimension to social isolation mentioned by participants outside of HIV clinical care involved internal feelings of isolation due to living with HIV. A participant discussed the dynamic of actually feeling less isolated as more people realized what it meant to live with a virus.

“Yeah, it triggers [the question] – are you positive? You’re like, for COVID? Or for [HIV] – so, that is very triggering…These days it makes me feel better, honestly, because people are starting to understand diseases and viruses more now. Like, they’re having open discussion about it. They’re having [to deal with] shit like that. (Yeah.) This has given me more peace of mind because now it’s – you’re not hiding it. COVID has so sadly helped with the mental [health]. You are talking about viruses now. People are talking about being positive now with their doctors. People are taking vaccines now. Six months ago, if you said positive, everyone froze like the fucking plague. I hate to say that, but COVID has helped.” (Participant 20, 30 years, cisgender man, Other Race - “Biracial,” Non-Hispanic/Latino, *Appraisal of relationships – emotional*)

Shared experiences of dealing with COVID-19 lessened feelings of social and mental isolation for this participant who actually found solidarity in others beginning to understand dealing with a viral illness like HIV. This fits the *appraisal of emotional relationships* domain in a sense, which was echoed by other participants who also noted feeling relief from knowing the whole country was confronting the same fears about living with an infectious disease, passing it on to others, visiting their doctors, and dealing with the implications of long-term morbidity and mortality.

## Discussion

The early COVID-19 pandemic presented unique challenges to the epidemic of social isolation faced by various communities, particularly PWH. PWH may have had heightened fears of COVID-19 due to their underlying immunocompromised state and the potential for re-traumatization of acquiring a new infectious disease – contributing to exacerbation of social isolation and loneliness. In our cohort, participants discussed a loss of contact with peers as a result of HIV stigma, expressed feeling disconnected from family and friends due to HIV status non-disclosure and/or anticipation and experience of HIV stigma. These feelings were exacerbated during the COVID-19 pandemic, a finding consistent with a recent study by Winwood et al., which showed that social isolation and loneliness was a key concern for PWH during COVID-19 across a range of studies [14].

The quality of HIV care during the early COVID-19 pandemic remained high among our participants due to a few factors, including streamlined in-person care and the use of telehealth as an effective supplement to in-person visits. Yet it was the provider-patient relationship that was the most important theme that emerged as a major facilitator to care continuity. Various studies in the literature have documented that the patient-provider relationship is crucial for HIV care engagement [11, 15–18], and this study extends this by providing qualitative evidence of its role as a potential protective factor during a public health emergency.

Furthermore, the multidisciplinary HIV care center played a critical role in mitigating various dimensions of social isolation during the pandemic. Elements of HIV clinical care provided benefits in 3 out of 5 domains of social isolation, and the *emotional appraisal of relationships* domain was the most frequent. Furthermore, it was different members of the multidisciplinary care team – in some cases the doctor, the nurse or phlebotomist for example – who each played important roles for different participants. It is unclear in the literature how social isolation impacts HIV care engagement more broadly [19], however our findings underscore the multi-dimensional *social benefit* a multidisciplinary HIV care center can provide. Given that the ongoing COVID-19 pandemic has increased social isolation across the board, as noted in the Surgeon General’s Advisory on the Healing Effects of Social Connection and Community, understanding the facets of HIV care that increase connectedness is an opportunity to support PWH and mitigate social isolation [3].

Continued in-person clinical care provided a wide array of social benefits in our study population, including the ability, as our participants noted, to see familiar faces, become educated about the COVID-19 pandemic, and speak candidly and openly about their health and reservations about vaccination. While telehealth provided some benefit, our cohort noted there were other benefits of in-person care that telehealth could not replace. This is in line with previous studies that have noted the limitations of telehealth in HIV care [20, 21]. The frequency and timing of contact via telehealth provided a sense of being cared for, and served as an alternative yet effective form of social support.

Taken together, the above findings underscore the need to understand and more intentionally replicate the aspects of social support provided to PWH in the flow of HIV clinical care. Emphasis should be given to the social support providers offer to patients via their consistent and intentional presence in the patient’s life and how telehealth can emulate the social support that in-person care provides (e.g., unannounced check-ins with patients via messaging in the electronic medical record app). Disruptive events like the COVID-19 pandemic can further isolate underserved groups and amplify already-existing feelings of loneliness and isolation. Interestingly our participants pointed to the feeling of solidarity they felt and lessened feelings of isolation having HIV and seeing others deal with the vulnerability of living through the COVID-19 pandemic.

An example of a specific service of the multidisciplinary care center that impacted social isolation was targeted outreach of patients by care coordinators and community health workers, which maintained a level of connectedness for some participants. These efforts have been strengthened moving by creating community among PWH through the care center in the form of consumer wellness groups where patients can support each other during challenging times. Consumer wellness groups, where patients can come together and share experiences about living with HIV, have the potential to mitigate social isolation and provide patient empowerment. For example, multiple research studies find that older adults with HIV are challenged by increased levels of loneliness and social isolation. Fewer than 20% of PWH have a partner or spouse and 70% live alone. The vast majority are Medicaid-dependent and are not employed [22]. Their social support networks are inadequate to meet the twin challenges of aging and HIV [23, 24]. The multidisciplinary care center has facilitated a clinic-based community of older peers with HIV for support, which has been successful. In our sample, having multiple team members reach out to check in on patients was effective; however, moving forward it may be effective to facilitate consumer wellness groups for all PWH during a crisis like the COVID-19 pandemic.

An important limitation to this study is that all patients were retained in care through COVID-19, introducing inherent bias into our sample as the voices of those out of care were not captured in this cohort. A more well-connected cohort at baseline may limit the generalizability of our findings. However, it is important to understand the facilitators of care continuity and the ways in which this group understands, perceives, and overcomes social isolation during COVID-19, and to consider ways of applying these lessons in other healthcare contexts. Future research may include cross-clinic comparisons to increase generalizability. Another limitation of our study is that, given this was a secondary analysis of a larger cohort study, we did not ask specific sociodemographic questions that would have better characterized our sample’s level of social isolation in Table 1 (e.g. living arrangement during COVID-19, number of romantic partners).

In conclusion, our study shows that in times of national upheaval like the COVID-19 pandemic, PWH may be particularly vulnerable to social isolation and the multidisciplinary HIV care model provides a potential foundation to mitigate the effects of social isolation on their overall health and HIV-specific outcomes (e.g. viral suppression and retention in care).
